# Germline Whole-Genome Sequencing in Early-Onset Pediatric Solid Tumors Implicates Novel Risk Factors

**DOI:** 10.1200/PO-26-00032

**Published:** 2026-06-03

**Authors:** Matthew Nagy, Andy Bhattacharjee, Abigail Cinelli, David Housman, Junne Kamihara, Jaclyn Schienda, Kayla Hamilton, Carrie Cibulskis, Christopher Ng, Noah Fields, Baihe Sun, Ryan Collins, Richard B. Parad, Lisa Diller, Riaz Gillani

**Affiliations:** ^1^ Department of Pediatrics, Boston Children's Hospital/Harvard Medical School, Boston, MA; ^2^ Broad Institute of Massachusetts Institute of Technology and Harvard University, Cambridge, MA; ^3^ Division of Newborn Medicine, Department of Pediatrics, Brigham and Women's Hospital/Harvard Medical School, Boston, MA; ^4^ Dana-Farber/Boston Children's Cancer and Blood Disorders Center/Harvard Medical School, Boston, MA; ^5^ Department of Biology, Massachusetts Institute of Technology, Cambridge, MA

## Abstract

**PURPOSE:**

Many children with very early-onset solid tumors remain without an identified germline risk factor after negative panel testing. Whole-genome sequencing (WGS) enables detection of large structural variants (SVs) and rare loss-of-function variants in highly constrained genes that are not routinely captured by standard clinical assays. The additional yield and spectrum of germline findings identified by WGS in this population remain incompletely defined.

**METHODS:**

We conducted a retrospective cohort study of children with very early-onset solid or brain tumors evaluated at a tertiary cancer genetic risk clinic with clinically guided germline panel testing. Germline WGS was performed on blood-derived DNA. Analyses focused on pathogenic or likely pathogenic variants in established cancer predisposition genes (CPGs), large SVs (>1,000,000 bp), aneuploidies, and loss-of-function variants in highly constrained genes.

**RESULTS:**

One hundred thirty-two patients were included, with median (IQR) age at diagnosis of 1.7 (0.8-3.2) years. Panel testing identified pathogenic CPG variants in 27 of 132 patients (20%). WGS recapitulated panel findings and identified nine additional putative pathogenic variants, increasing yield to 27%. Eight patients (6%) harbored large germline SVs or aneuploidies, including five events not previously recognized. Rare loss-of-function variants in highly constrained genes were identified in 46 patients (35%), many involving pathways relevant to cancer development. Overall, 66 of 132 patients (50%) carried at least one rare germline variant of potential pathogenic relevance.

**CONCLUSION:**

In children with very early-onset solid tumors, germline WGS increased detection of potentially pathogenic variants, including novel structural and constrained-gene alterations. These findings support broader consideration of germline WGS in early-onset solid tumors to refine genetic risk assessment and enable discovery of novel susceptibility mechanisms.

## INTRODUCTION

Germline pathogenic variants in established cancer predisposition genes (CPGs) account for approximately 8%-10% of unselected childhood cancers, with higher rates reported in some solid tumor subtypes.^1^ Although there is evidence that children with very early-onset tumors have a higher rate of pathogenic CPG mutations,^2^ such as in rhabdomyosarcoma,^3^ the majority of these patients undergo panel-based germline testing without identification of an actionable predisposition variant. Whole-genome sequencing (WGS) enables detection of variant types not captured by panel testing, including large structural variants (SVs) and rare loss-of-function variants in genes that are highly constrained against variation in the general population.^4^

**CONTEXT**

**Key Objective**
What additional potential germline risk factors can be identified when whole-genome sequencing (WGS) is used to evaluate children with very early-onset solid tumors?
**Knowledge Generated**
In this study, germline WGS of children with very early-onset solid tumors identified rare predicted loss-of-function variants in both established cancer predisposition genes, along with highly constrained genes and large structural variants not routinely included in clinical testing panels. Approximately half of the patients harbored at least one rare germline variant of potential relevance, many of which involved pathways inherently relevant to cancer biology.
**Relevance**
These findings suggest that germline WGS may expand the landscape of candidate cancer susceptibility genes and highlight biologic pathways that could contribute to inherited risk in pediatric cancer.

We applied WGS to a clinically enriched cohort of pediatric patients with very early-onset solid tumors who had previously undergone multigene germline panel testing, with the goal of (1) evaluating the effectiveness of WGS in identifying previously identified panel genes (given lower sequencing depth) and (2) identifying additional classes of germline variation that may contribute to cancer risk beyond known CPGs.

## METHODS

### Cohort and Prior Germline Testing

We identified 132 children who were evaluated in the Dana-Farber Cancer Institute Pediatric Cancer Genetic Risk Clinic and had a history of a solid or brain tumor at age <9 years. Patients underwent clinical evaluation previously, including targeted panel testing at the discretion of the care team. Written informed consent was obtained for participation in research studies, which included WGS and data-sharing. The study was approved by the Dana-Farber Institutional Review Board. Clinical panel results were abstracted as positive (pathogenic/likely pathogenic variant detected) or negative/variant of unknown significance, and clinical data were obtained from medical record review.

### Whole-Genome Sequencing, Variant Calling, and Annotation

Germline WGS (approximately 30×) was performed on blood-derived DNA using the Illumina NovaSeq 6000 or NovaSeq X platform. Cluster amplification and 151 bp paired-end sequencing were conducted with Illumina sequencing-by-synthesis chemistry, and raw data were processed through the Picard/Zamboni pipeline to generate aligned CRAM files. Variant calling was performed using the DRAGEN Bio-IT Platform (v3.2.8) for single-nucleotide variants (SNVs) and small insertions/deletions (indels), and SVs > 50 bp were identified using the DRAGEN CNV pipeline and Manta v1.4.0. SNVs/indels were retained if they were (1) rare in the general population (allele frequency <1% in gnomAD) and (2) predicted to have a loss-of-function annotation with Variant Effect Predictor by Ensemble and/or annotated pathogenic in ClinVar. Deletion SVs were retained if they were rare in our cohort (<1%), as there was no comprehensive population database to compare. Alterations involving only introns or the terminal exon were excluded. Rare large SVs >1,000,000 bp (both deletions and duplications), including aneuploidies, were retained. Aneuploidies were predicted based on the contig-level ploidy estimates generated by GATK-SV; specifically, we retained any predicted aneuploidy corresponding to a whole-contig copy-number estimate <1.5 or >2.5 copies for monosomy or trisomy, respectively, with corresponding adjustments on allosomes to handle sex ploidy differences between males and females. All SNVs/indels and SV calls were manually reviewed using the Integrative Genomics Viewer and/or read depth plots to filter out false-positive calls.

Three classes of germline variation were assessed:

1. Established CPGs (manually curated list of 148 genes)^5-8^
2. Large SVs (deletions, duplications) and constitutional aneuploidies, and
3. Variants in highly constrained genes (top sextile of loss-of-function intolerance identified from gnomAD version two resulting in a list of 3,010 genes).^9^

To evaluate potential combinatorial effects between constrained gene variants and pathogenic or likely pathogenic variants in known CPGs, patients were categorized into four groups: CPG variants only, constrained gene variants only, both variant types, or neither. Age at diagnosis was compared across groups using a Kruskal-Wallis test.

### Reactome Pathway Analysis

To evaluate whether constrained genes harboring predicted loss-of-function variants converged on biologically relevant pathways, we performed Reactome pathway analysis using the ReactomePA R package with all protein coding genes as the background gene set. *P* values comparing constrained genes with mutations of interest to all coding genes were calculated using a hypergeometric test and adjusted for multiple testing, and an unadjusted *P* value of <0.05 was used to nominate genes for further pathway analysis. Pathways intersecting ≥2 identified genes were retained and were manually grouped in broad cancer-related categories (RTK/growth signaling/developmental signaling, chromatin/post-translational or transcriptional regulation, DNA damage/genome stability) or not canonically cancer-related by literature review.

### External Cohort Interrogation

To evaluate whether constrained genes identified in our cohort were also observed in independent germline pediatric cancer data sets, we queried the Gabriella Miller Kids First Data Resource Center (GMKF) variant portal for constrained gene variants that appeared to have relevance to cancer pathways based on Reactome Analysis. Variants were filtered to include rare (gnomAD allele frequency <0.01) and those predicted to be loss-of-function (stop-gain, frameshift, and canonical splice-site variants). Searches were restricted to pediatric cancer studies available in the GMKF portal with germline data, and the presence of qualifying variants across studies was recorded to provide supporting evidence from independent pediatric germline whole-genome sequencing cohorts.

## RESULTS

**FIG 1.**
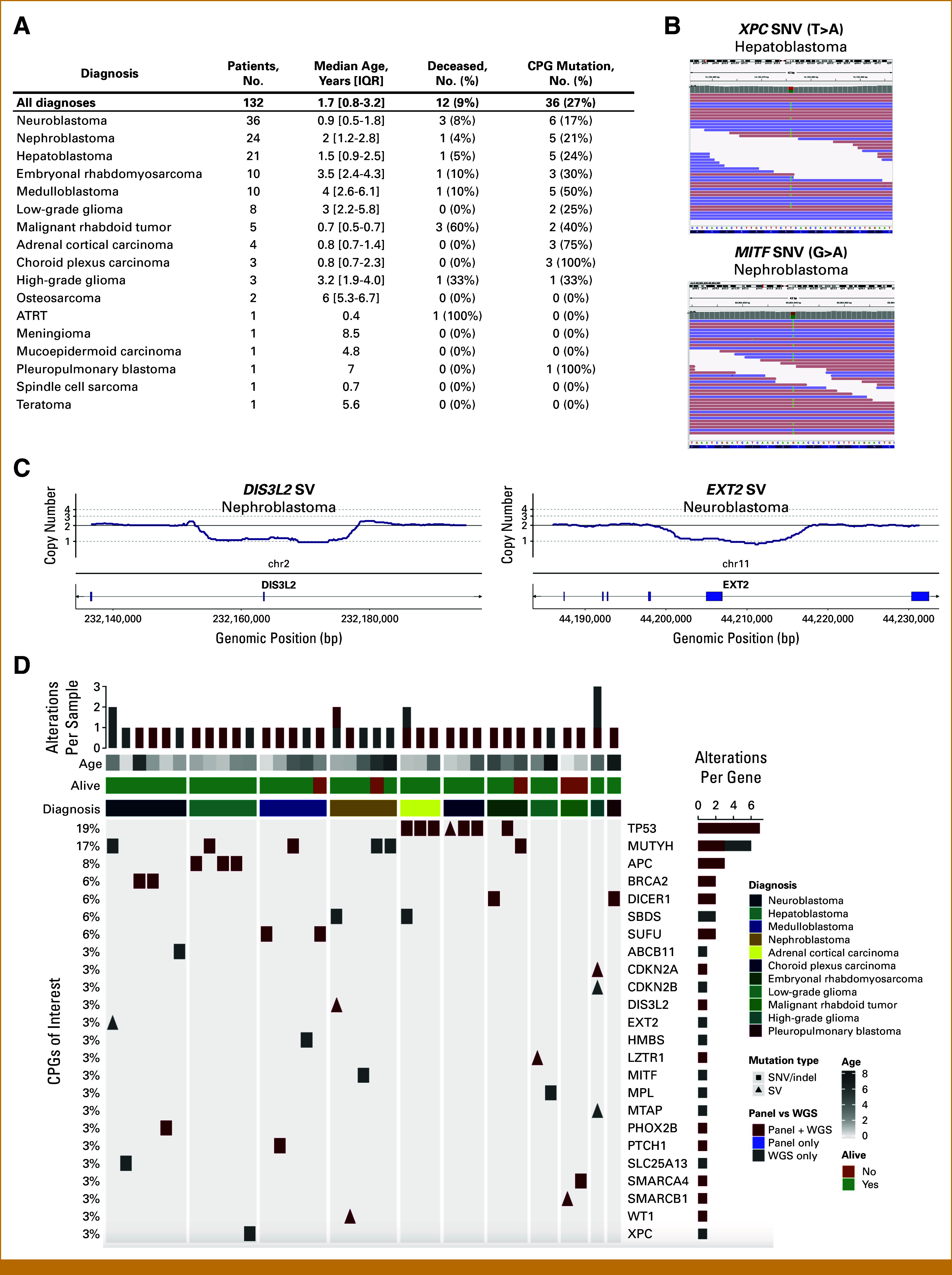
Patient characteristics and CPGs: (A) Patient characteristics. (B, C) Example validation snapshots of (B) SNV/indels on the IGV and (C) SVs on read depth plots. (D) Oncoprint of germline alterations of known CPGs found on panel sequencing and whole-genome sequencing. ATRT, Atypical teratoid rhabdoid tumor; CPG, cancer predisposition gene; IGV, interactive genome viewer; SNV, single-nucleotide variant; SV, structural variant; WGS, whole-genome sequencing.

Overall, 132 patients were included, with the most common cancers including neuroblastoma (27%), nephroblastoma (18%), and hepatoblastoma (16%), reflecting the referral patterns of solid tumor/brain tumor patients to the study. The median [IQR] age at diagnosis was 1.7 [0.8-3.2] years (Fig 1A).

### Known CPG Variants

Germline panel testing identified pathogenic or likely pathogenic CPG variants in 27 (20%) of 132 patients, spanning 14 CPGs including high penetrance genes such as *TP53* (n = 7), *DICER1* (n = 2), *WT1* (n = 1), and *SMARCB1* (n = 1). WGS detected all 27 panel-identified events and identified additional putative pathogenic CPG variants in nine more patients including SNVs in *MUTYH* (n = 3; hepatoblastoma, neuroblastoma, nephroblastoma), *SBDS* (n = 2; hepatoblastoma, neuroblastoma), *HMBS* (medulloblastoma), *MITF* (nephroblastoma), *MPL* (low-grade glioma), *SLC25A1* (neuroblastoma), *XPC* (hepatoblastoma), and an SV affecting *EXT2* (neuroblastoma; Figs 1B-1D). The addition of WGS resulted in a total of 36/132 (27%) patients with a germline pathogenic variant in an established CPG.

### Large SVs and Aneuploidies

**FIG 2.**
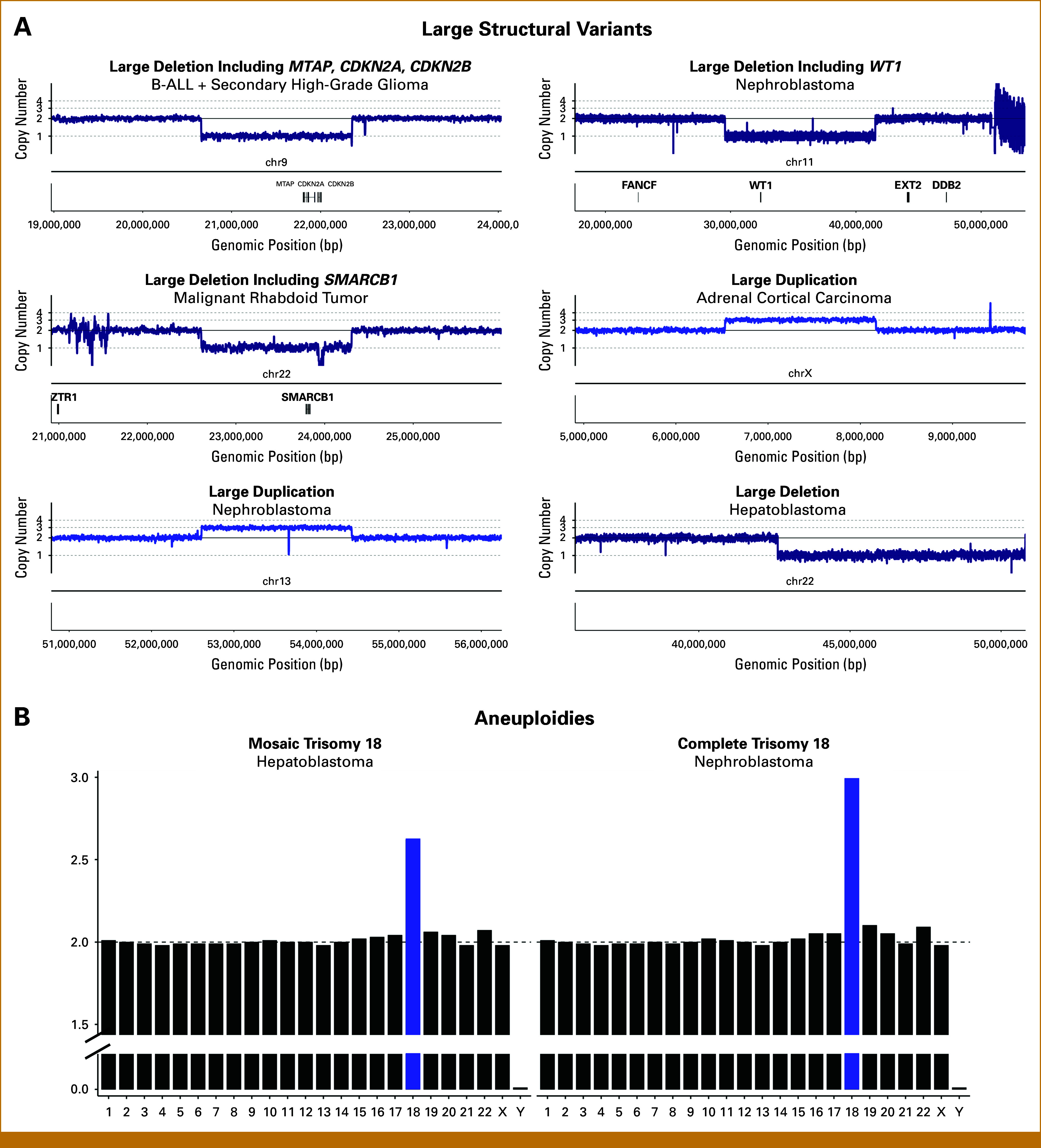
Large SVs and aneuploidies: (A) Read depth plots of all identified large (>1,000,000 bp) SVs and (B) chromosomal copy-number plot highlighting aneuploidies. Gene tracks only include CPGs, constrained genes excluded, given space limitations. CPG, cancer predisposition gene; SV, structural variant.

WGS identified large germline alterations (six large SVs, two aneuploidies) in eight patients (6%; Fig 2A). Three involved established CPGs: a *SMARCB1* deletion in a patient with malignant rhabdoid tumor, a *CDKN2A/B/MTAP* codeletion in a patient with a high-grade glioma (after a prior diagnosis of B-ALL), and a *WT1*/11p13 deletion in a patient with nephroblastoma and WAGR syndrome. Of these, only the *WT1* deletion had been identified in panel testing as a large SV; the *SMARCB1* and *CDKN2A/B/MTAP* events had been reported only as single-gene findings on panel testing. The remaining three large SVs, one deletion and two duplications, were not detected by prior testing (Fig 2A).

Two constitutional aneuploidies were observed: complete trisomy 18 in a patient with nephroblastoma and mosaic trisomy 18 in a patient with hepatoblastoma (Fig 2B). Both were known clinically from prior clinically indicated karyotype testing due to neonatal phenotype.

### Variants in Constrained Genes

**FIG 3.**
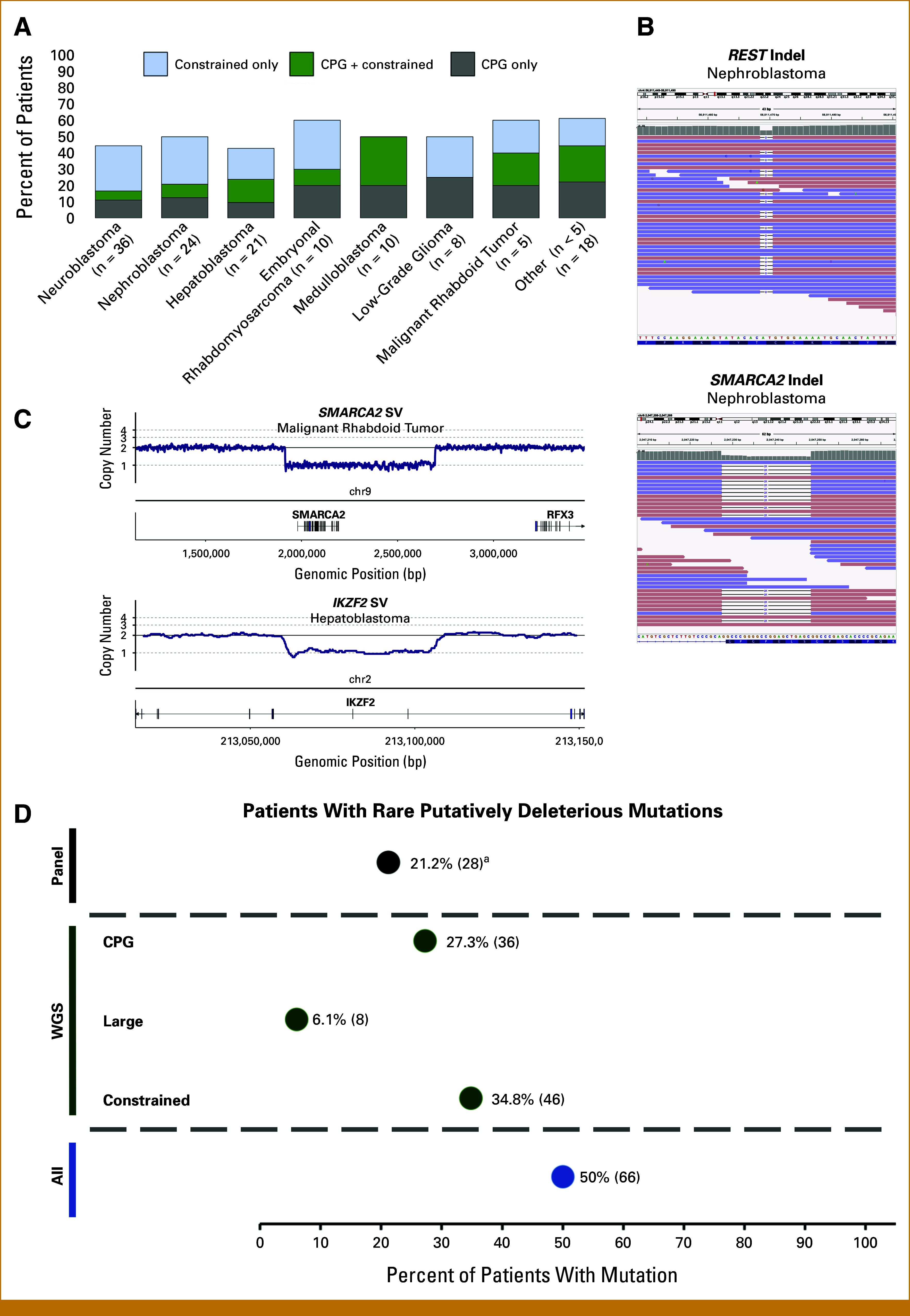
Constrained genes and summary: (A) Percentage of patients with a constrained gene mutation, CPG mutations, or both across cancer types. Cancers with less than five samples were grouped together. (B, C) Example validation snapshots of (B) SNV/indels on the IGV and (C) SVs on read depth plots. (D) Summary of patients identified with rare putatively deleterious germline mutations on panel sequencing, WGS of known CPGs, large SVs, and constrained genes, and number of patients with at least one WGS alteration. ^a^Twenty seven panel genes identified were CPGs and one was a constrained gene (KMT2D) identified in evaluation for suspected Kabuki syndrome. CPG, cancer predisposition gene; IGV, interactive genome viewer; SNV, single-nucleotide variant; SV, structural variant.

Across the cohort, 80 rare high-impact variants (46 SNV/indel and 34 SV) in constrained genes were identified in 46 patients (35%; Fig 3A). Notably, 21 of the constrained genes identified were within large SVs (Appendix Table A1). In 30 patients (23%), a constrained-gene variant was the only rare germline finding; in 16 patients (12%), it co-occurred with a CPG variant, a pattern that was largely consistent across cancer types (Fig 3A; Appendix Table A1). There was no significant difference in median age [IQR] of onset between patients with variants in constrained genes only (1.7 [2.0] years), CPG only (2.3 [2.8] years), both CPG and constrained (1.3 [2.7] years) and neither (1.7 [2.1] years, *P* = .41). Notable constrained-gene variants included *SMARCA2* (n = 2; rhabdoid tumor, nephroblastoma), *EIF4G1* (n = 2; choroid plexus carcinoma, mucoepidermoid carcinoma after a prior diagnosis of AML), *REST* (nephroblastoma) and *FGFR1* (neuroblastoma; Fig 3B). A complete list of constrained genes with detected high-impact variants is provided in Appendix Table A1.

**FIG A1.**
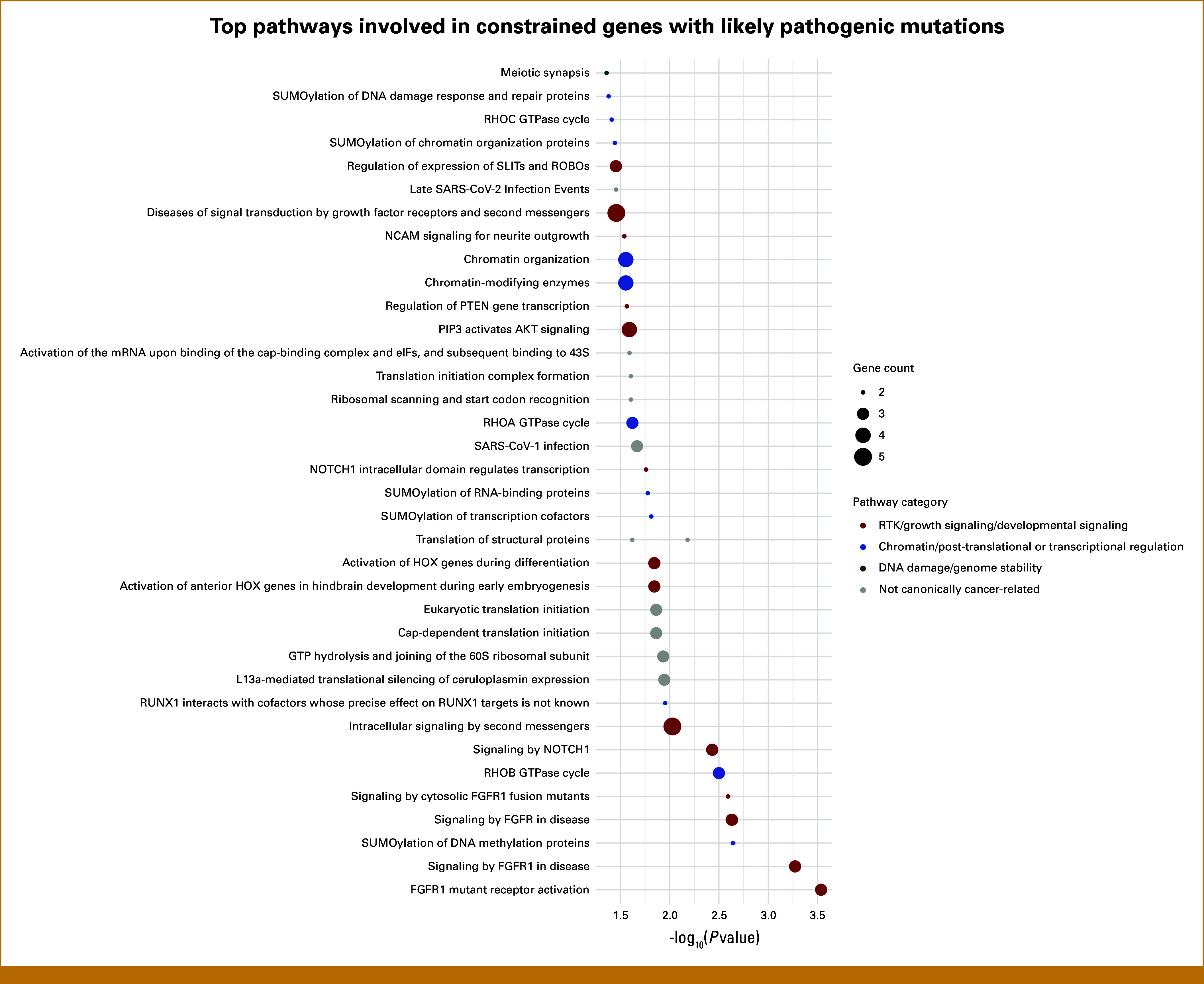
Reactome pathway analysis of identified loss-of-function–constrained gene variants and overlap with cancer-relevant pathways.

Reactome Pathway analysis (see Methods section) led to the identification of a subset of constrained genes related to biologically relevant pathway themes (Appendix Fig A1); the following 23 genes were found to intersect with cancer relevant pathways across 20 distinct patients*: ADAM10, AP3B1, BCR, CBX2, DLC1, EIF4G1, FGFR1, HIF1A, KMT2D, MYO9A, NBEA, NCOA3, PAX6, PTK2, REST, ROBO2, RPL4, SMARCA2, SMC1B, TRAF6, TRRAP, UBE2I, ZMYM2*. Of note, 17 of the 23 constrained genes found to be related to cancer-associated pathways were described to have rare loss-of-function variants across multiple samples and cancer types in external pediatric cancer GMKF data sets, 11 of which occurred without a concurrent panel identified CPG mutation, providing additional evidence for potential relevance for their role in disease pathogenesis (Appendix Table A2).

### Combined Germline Contribution

Considering all three variant classes: established CPGs (27%), large SVs (6%), and constrained-gene variants (35%), 50% (66 of 132) of patients carried at least one rare germline variant of potential relevance (Fig 3C). All identified mutations along with patient characteristics, phenotypic information, and family cancer history are available in Appendix Table A1. Of note, 12 cases had a history of more than one tumor by the time of enrollment in the study, in these, the germline contribution was 83% (10/12), with 66% (8/12) identified on panel sequencing.

## DISCUSSION

In this clinically enriched cohort of children with very early-onset solid tumors, germline panel testing identified pathogenic variants in established CPGs in 20% of patients, a rate higher than the approximately 8-10% reported in unselected pediatric cancer populations,^1^ consistent with prior reports of increased CPG detection in children with younger cancer onset.^2^ WGS replicated these panel findings and increased the rate of identification of putatively pathogenic CPGs mutations to 27% of patients, although the relationship between some of these additional identified CPG variants and the occurrence of childhood cancers is not yet established. Prior work in whole-exome sequencing, however, has demonstrated improved detection of actionable mutations compared with targeted panels in pediatric oncology,^10^ and our work underscores the possible value of broader testing when clinical suspicion for heritable risk is high.

Beyond known CPGs, WGS revealed five previously unreported (clinically unrecognized) large SVs, two of which clarified the structural context of two additional gene-level findings that appeared as single-gene mutations on panel testing, highlighting how WGS can resolve SVs that standard assays cannot detect. Recent large-scale analyses similarly demonstrate that rare germline SVs account for an estimated 1%-5% of pediatric cancer predisposition, particularly in the youngest-onset cases.^11^

WGS also identified rare, predicted loss-of-function variants in highly constrained genes in 35% of patients, including variants in *SMARCA2* in patients with rhabdoid tumor and nephroblastoma, a gene in the same SWI/SNF family as the canonical rhabdoid predisposition genes,^12^ and *REST* in a child with nephroblastoma, a gene well established as a somatic variant in nephroblastoma,^13^ with early evidence suggesting germline relevance.^14^ Although most constrained-gene variants are not yet clinically actionable, they nominate biologically plausible candidates for future case-control studies and functional validation. Importantly, many of the identified mutated constrained genes reflect pathways known to be associated with cancer such as chromatin-modifying enzymes, cap-dependent translation initiation, and signaling by *FGFR1* and *NOTCH1,* and similar loss-of-function mutations were identified in public pediatric cancer cohorts, consistent with other known CPGs that are evolutionarily constrained.^15,16^

Taken together, 50% of patients carried at least one rare germline pathogenic variant of potential relevance across CPGs, large SVs, or constrained genes, suggesting that inherited susceptibility in the earliest-onset pediatric cancers may be substantially underestimated when relying on panel-based testing alone. An important limitation to note, however, is that patients were recruited from a cancer genetics clinic at a tertiary care center, a selection bias that likely contributed to the high overall rate of pathogenic germline variants detected. These data support a tiered approach to germline evaluation in pediatric oncology, in which WGS is considered for patients with early age at diagnosis, atypical tumor type, or negative panel testing. Larger case-control studies are needed to define penetrance, pathogenicity, and clinical utility of emerging variant classes.

## APPENDIX

**TABLE A1.** Patient-Level Demographic, Mutation, Phenotypic, and Family History Data

Patient	Cancer	Race	Age, Years	Panel	CPG_SNV_INDEL	CPG_SV	Constrained_SNV_INDEL	Constrained_SV	Large SV (Chr, BP)	Aneuploidy	Phenotype	Family History
17104_000134_090319	Hepatoblastoma	Other	1.3	APC	APC		NBEA				Negative	Mother and maternal grandmother with FAP
17104_000453_070920	Hepatoblastoma	White	0.7	APC	APC		SLC41A2				Developed colon adenocarcinoma at age 12 years. Congenital nephrotic syndrome with ESRD. Renal and liver transplant at age 1 year	Mother APC carrier, paternal grandmother and great maternal grandmother with breast cancer in 40s/50s
17104_000853_011223	Hepatoblastoma	Unknown	2	APC	APC			IKZF2			Negative	Maternal grandmother with brain cancer, maternal aunt with BRCA1/2 mutation, father with colon polyps
17104_000432_012320	Neuroblastoma	White	1.7	BRCA2	BRCA2		ADAM10, HSPA12A, MIA3				Negative	Paternal grandmother with breast cancer at age 38 years, maternal great uncle with pancreatic cancer
17104_000341_092019	Neuroblastoma	White	6.2	BRCA2	BRCA2						Hypospadias at birth. Later developed papillary thyroid carcinoma	Maternal grandfather with prostate cancer at age 70 years, maternal uncle with low-grade astrocytoma at age 13 years, paternal grandmother with breast cancer at age 71 years, paternal grandfather with papillary thyroid carcinoma at age 68 years
RP-2006_PROCA790-DNA1_v1_WGS_GCP	High-grade glioma	White	3.2	CDKN2A		CDKN2A,^a^ CDKN2B,^a^ MTAP^a^			chr9 DEL, 1,688,937 bp		Negative	Maternal grandfather with prostate cancer at age 60 years, maternal great grandmother with breast cancer at age 40 years, maternal great great uncle with leukemia, paternal grandmother with breast cancer at age 48 years
17104_000719_041522	Pleuropulmonary blastoma	White	7	DICER1	DICER1		CELSR3				Negative	Mother with DICER1 mutation, sister with DICER1 and Sertoli-Leydig tumor
17104_000274_051719	Embryonal rhabdomyosarcoma	Black or African American	4.4	DICER1	DICER1			ATG13			Negative	Mother with brain tumor at age 14 years, recently deceased, maternal aunt with recent death, maternal grandmother with breast cancer
17104_000482_072120	Nephroblastoma	White	1.1	DIS3L2	SBDS	DIS3L2					1 cm freckle on hand, one small pigmented lesion on scalp	Maternal grandfather with esophageal cancer at age 46 years
PROCA697-DNA1	Hepatoblastoma	White	2.6	KMT2D			ATP2C1, KMT2D				Kabuki syndrome, 31-week preemie, tetralogy of fallout, neonatal hypoglycemia, slower with walking development, low-set ears, asymmetric small right kidney and left kidney with duplicated collecting system with pelviectasisand a small cyst	Paternal aunt with ovarian cancer at age 17 years, paternal great uncle with leukemia in 70s, maternal grandfather with colon cancer, maternal cousin with Hodgkin lymphoma at age 12 years
PROCA989-DNA1	Low-grade glioma	White	2.2	LZTR1		LZTR1					30w preemie, IUGR, initial torticollis. Early intervention for speech delay, hypospadias, and high-riding left testicle	Maternal grandfather with lung cancer at age 70 years
PROCA854-DNA1	Medulloblastoma	White	3.8	MUTYH	MUTYH			TRPS1			High palate, history of dyspraxia, mild thoracolumbar kyphosis and mild thoracolumbar endplate degeneration, larger joints in hands and thumb, benign pigmented nevi	Maternal grandmother with breast cancer in 70s, maternal aunt with breast cancer in 40s, paternal grandfather with prostate cancer in 50s, three paternal great uncles with prostate cancer, two paternal aunts with breast cancer in 50s, one paternal uncle with lung cancer in 50s
17104_000302_030620	Hepatoblastoma	Other	0.9	MUTYH	MUTYH					Mosaic trisomy 18, CN 2.62	Mosaic trisomy 18, fetal growth restriction, seizures, early pyelonephritis	Paternal aunt with unspecified cancer
17104_000760_051622	Embryonal rhabdomyosarcoma	Unknown	4	MUTYH	MUTYH						Pink birthmark on neck and small tan birthmark on neck	Maternal grandmother with non-Hodgkin lymphoma in 40s, paternal grandfather with bladder cancer in 50s
17104_000781_090722	Neuroblastoma	Unknown	0.5	PHOX2B	PHOX2B						Central hypoventilation, hypoxic ischemic injury, dysautonomia	Negative
17104_000614_092221	Medulloblastoma	Unknown	1.2	PTCH1	PTCH1		NCOA3				Palmer pits, odontogenic keratocysts, multiple early basal cell carcinomas, macrocephaly, mild hypotonia, mild to moderate ataxia, multiple absent teeth	Paternal grandmother with ovarian cancer
RP-2006_PROCA784-DNA1_v1_WGS_GCP	Malignant rhabdoid tumor	White	0.7	SMARCA4	SMARCA4						Negative	Maternal great grandmother with breast cancer at age 35 years, maternal cousin with ovarian surgery at age 15 years
17104_000533_031821	Malignant rhabdoid tumor	Unknown	0	SMARCB1		SMARCB1^a^		BCR,^a^ GNAZ,^a^ SPECC1L^a^	chr22 DEL, 1,694,444 bp		Prenatal diagnosis of rhabdoid tumor	Negative
17104_000369_020720	Medulloblastoma	White	0.2	SUFU	SUFU		EIF3F				Later developed benign nerve sheath tumor, dysplastic nevus, maxillary sinus spindle cell sarcoma, testicular leiomyoma, atrial septal defect	Mother with colon cancer age 42 years (mother adopted)
RP-2006_PROCA750-DNA1_v1_WGS_GCP	Medulloblastoma	Not reported	2.2	SUFU	SUFU						Impacted teeth, upper-extremity pigmented lesions	Maternal grandmother with ovarian and breast cancer in 70s, father with pancreatic adenocarcinoma in 60s
RP-2006_PROCA888-DNA1_v1_WGS_GCP	Adrenal cortical carcinoma	White	0.7	TP53	SBDS, TP53		AP3B1, HIF1A				Negative	Maternal great aunt with melanoma and another with lung cancer in 40s/50s
17104_000821_102622	Adrenal cortical carcinoma	White	3.2	TP53	TP53		SPTY2D1				Negative	Siblings without cancer but with TP53 mutation, father with Li-Fraumeni syndrome and leukemia at age 9 years and melanoma at age 38 years, paternal grandmother with breast cancer at age 37 years, paternal grandfather with history of brain tumor, paternal aunt with brain tumor at age 9 years, paternal uncle with brain tumor at age 30 years
17104_000275_120319	Adrenal cortical carcinoma	White	0.8	TP53	TP53						Negative	Mother with osteoma at age 32 years, maternal grandmother with thyroid nodule in 40s, maternal grandfather with prostate cancer in 50s, maternal great uncle with colon cancer in 50s, maternal great aunt with breast cancer in 30s. Father with osteosarcoma at age 17 years and colon cancer at age 36 years, paternal great uncle with melanoma at age 56 years
17104_000596_082521	Choroid plexus carcinoma	White	0.8	TP53	TP53						Cyst on brain in utero, macrocephaly, and large for gestational age	Maternal great grandmother with breast cancer in 70s, maternal aunt with ovarian cancer, maternal great aunt with breast cancer at age 45 years, maternal half aunt with breast cancer at age 44 years, father with TP53mutation but no cancer, paternal grandmother with breast cancer at age 35 years and neuroendocrine carcinoma at age 49 years, paternal great grandfather with breast cancer, paternal grandfather with leukemia at age 53 years, and great aunt with breast cancer
17104_000629_080422	Choroid plexus carcinoma	White	3.8	TP53	TP53						Negative	Both parents negative for TP53
17104_000665_050723	Embryonal rhabdomyosarcoma	White	2.4	TP53	TP53						Later developed renal cell carcinoma	Both parents negative for TP53
17104_000232_021821	Choroid plexus carcinoma	White	0.5	TP53		TP53	EIF4G1				Negative	Paternal grandfather with colon cancer at age 70 years and paternal grandmother with breast cancer at age 60 years
17104_000202_061621	Nephroblastoma	White	1.2	WT1		WT1^a^	PTK2	CAPRIN1,^a^ CSTF3,^a^ FBXO3,^a^ FJX1,^a^ HIPK3,^a^ KCNA4,^a^ LRRC4C,^a^ MPPED2,^a^ PAX6,^a^ QSER1,^a^ TRAF6^a^	chr11 DEL, 11,986,466 bp		WAGR, relapsed Wilms	Negative
17104_000915_031723	Neuroblastoma	Unknown	1.6		ABCB11						Negative	Negative
PROCA924-DNA1	Medulloblastoma	White	4.2		HMBS						In vitro fertilization twin, low muscle tone, and cataplexy	Negative
17104_000401_081220	Nephroblastoma	White	3.6		MITF						Beckwith-Wiedemann syndrome, large tongue at birth through first year, umbilical hernia, heart murmur, right arm and leg larger than left	Maternal great grandmother with breast and lung cancer in 50s, maternal great aunt with Hodgkin lymphoma at age 18 years, maternal great uncle and aunt with lung cancer
PROCA993-DNA1	Low-grade glioma	White	7.7		MPL						Negative	Maternal great uncle with brain cancer and skin cancer
17104_000594_062821	Neuroblastoma	White	2.9		MUTYH	EXT2					Negative	Negative
17104_000783_072222	Nephroblastoma	White	5.7		MUTYH		RELN		chr13 DUP, 1,821,141 bp		Negative	Negative
PROCA913-DNA1	Nephroblastoma	White	6.9		MUTYH						Negative	Maternal grandmother with cervical cancer, paternal uncle with thyroid cancer at age 32 years
17104_000147_060718	Neuroblastoma	White	0.2		SLC25A13		TRRAP				Negative	Negative
17104_000893_021423	Hepatoblastoma	White	2.1		XPC						Negative	Paternal grandfather with colon cancer, father with polyps
RP-2006_PROCA824-DNA1_v1_WGS_GCP	Embryonal rhabdomyosarcoma	White	6.2				ANKS1B				Reddish birthmark 3-4 inches above left clavicle, later developed melanoma	Mother with breast cancer at age 48 years, maternal grandmother with leiomyosarcoma of colon at age 42 years, maternal grandfather with pancreatic cancer at age 51 years
17104_000648_011822	Hepatoblastoma	White	0.8				CACNA1B				Intrauterine growth restriction, tracheobronchomalacia, micropenis, hypospadias, bilateral inguinal hernias, congenital hypothyroidism	Maternal grandmother with cervical cancer at age 29 years
17104_000440_020822	Neuroblastoma	Other	0.2				CBX2	ROBO2			Negative	Maternal great grandmother with breast cancer at age 55 years
17104_000583_080221	Neuroblastoma	White	2.8				DDX27, ZNFX1				Low muscle tone at birth, choroidal nevus	Maternal grandmother with breast cancer at age 55 years, maternal uncle with glioblastoma at age 45 years, great grandmother with bladder cancer in 90s, great uncle with lung cancer in 60s
17104_000532_122220	Hepatoblastoma	Asian	3.5				DLC1				Negative	Negative
PROCA617-DNA1	Mucoepidermoid carcinoma	White	4.8				EIF4G1				AML at age 1 years, mucoepidermoid carcinoma at age 4 years with recurrence, cafe au lait on leg	Sister with cafe au lait spots, paternal great aunt with breast cancer, paternal great grandmother with breast cancer in 70s, maternal first cousin with breast cancer at age 36 years, maternal great grandfather with unknown cancer, maternal great grandmother with gynecologic cancer
17104_000612_011021	Neuroblastoma	Black or African American	2.4				FGFR1, GRHL3	UBE2I			Negative	Paternal grandmother with colon cancer
PROCA785-DNA1	Low-grade glioma	White	0.5				GANAB				Red birthmark on back of neck and two café au lait spots, spitz nevus, incidental pineal cyst found on imaging, recurrent fevers daily	Maternal great aunts and uncles with breast and colon cancer in old ages, maternal great grandmother with CLL at age 55 years, paternal great great grandmother with osteosarcoma in adulthood, paternal great grandfather with colon cancer, paternal great great grandmother with leukemia as an adult
17104_000035_061719	Neuroblastoma	White	1.7				LEPR				Two strawberry hemangiomas on head and spine	Maternal great aunt and great grandmother with breast cancer, paternal grandmother with papillary thyroid cancer, paternal great grandmother with leukemia, paternal great uncle with 18 colon polyps
17104_000426_041421	Neuroblastoma	White	0				LRRC7				Prenatally diagnosed with paraspinal mass, left femur fracture from birth trauma	Maternal great grandfather with melanoma and great uncle with lung cancer, paternal great grandmother and great great aunt with breast cancer in 50s
17104_000123_030222	Neuroblastoma	Other	1.3				LTN1	MYO9A			Negative	Negative
17104_000855_020223	Neuroblastoma	White	0.9				LYPLA2				Negative	Paternal grandmother and great aunt with lung cancer, both smokers, paternal grandfather with melanoma, paternal uncle with salivary gland tumor in 30s, maternal great grandfather with prostate cancer in 80s
17104_000497_060721	Nephroblastoma	White	2.7				MED12L				Negative	Maternal great aunt with lung cancer at age 50 years
PROCA836-DNA1	Embryonal rhabdomyosarcoma	Unknown	4.1				NMT1				Negative	Maternal uncle with prostate cancer at age 45 years
PROCA920-DNA1	Low-grade glioma	White	3.2				PPFIA3				Larger head at birth, low muscle tone	Maternal grandmother with cervical cancer at age 40 years, maternal great uncle with cancer at age 70 years, two maternal great uncles with pancreatic cancer in 60s/70s, maternal great aunt abdominal cancer at age 40 years, two paternal cousins with colon cancer at age 40 years and 60 years, paternal great uncle with leukemia at age 40 years
17104_000154_070919	Nephroblastoma	Asian	1.7				REST				Possible speech delay, one brown mark on buttocks	Maternal grandmother with uterine cancer at age 65 years, maternal great grandmother with pancreatic cancer, maternal great grandfather lung cancer, paternal uncle with unilateral Wilms tumor at age 2 years, paternal grandfather with lung cancer
PROCA1016-DNA1	High-grade glioma	White	0.6				REXO1				Small hyperpigmented spot on back size of dime, and small light spot on legs	Maternal great grandmother with throat cancer at age 82 years, maternal distant cousin with leukemia at age 11 years
17104_000374_031021	Nephroblastoma	Black or African American	1.1				RPL4, SPATS2				Slow walking development, two possible cafe au lait spots on shoulder/chest and left leg	Maternal great grandmother with kidney cancer in 40s
17104_000152_082219	Nephroblastoma	White	1.5				SMARCA2				Negative	Maternal uncle with ependymoma at age 17 months, paternal great grandfather and grandmother with lung cancer in 70s
17104_000148_070821	Neuroblastoma	White	0.2				TARDBP				Hemangioma on back of neck, right side undescended testicle	Maternal great uncle with skin cancer, maternal great grandmother lymphoma at age 90 years, maternal second cousin breast cancer in 50s, maternal great grandfather prostate cancer in 60s. Paternal grandmother with breast cancer at age 44 years, paternal great grandmother with colon cancer in 80s, paternal great grandfather with bladder cancer in 80s, paternal first cousin once removed with astrocytoma at age 10 years
17104_000746_042922	Nephroblastoma	Unknown	2.8				TSC22D4				One hyperpigmented birthmark on lower back	Maternal grandmother with breast cancer (HOXB13 variant), maternal grandfather with prostate cancer, maternal great aunt with breast cancer, maternal great uncle with prostate cancer, maternal first cousin once removed with breast cancer at age 36 years
17104_000753_050522	Embryonal rhabdomyosarcoma	White	3				ZMYM2				Stork bite on back of neck, astigmatism	Maternal great aunt and great grandmother with lung cancer, maternal grandfather with cancer in 50s and great grandfather with liver cancer in 70s, paternal great aunt and great grandmother with colon cancer in 50s and 80s
17104_000847_123022	Malignant rhabdoid tumor	White	0.7				ZNF24	SMARCA2			Negative	Negative
17104_000373_121819	Hepatoblastoma	White	2.5					CELSR1,^a^ FBLN1,^a^ GRAMD4,^a^ PHF21B,^a^ SCUBE1,^a^ SMC1B,^a^ SULT4A1^a^	chr22 DEL, 6,707,245 bp		Prenatal polycystic kidney, 22q13.3 deletion and 8p duplication, syndactyly, hypoplasia of corpus callosum, prematurity, intestinal perforation, retinopathy of prematurity	Maternal great grandfather with prostate cancer in 60s and stomach cancer in 80s, maternal great uncle with leukemia in 30s, maternal great aunt with breast cancer in 50s
17104_000376_110819	Adrenal cortical carcinoma	White	0.7					DDX4	chrX DUP, 1,628,396 bp		Negative	Maternal great aunt with breast cancer at age 52 years, maternal great grandmother with breast cancer. Paternal second cousin with bilateral Wilms tumor at age 7 years, paternal second cousin with testicular cancer at age 2 years
17104_000609_112321	Nephroblastoma	White	0.9					HORMAD1			Beckwith-Wiedemann syndrome, mild macroglossia, hydronephrosis in utero	Maternal great aunt with colon polyps, maternal great aunt with benign pulmonary tumor, paternal great uncle with leukemia in old age, and one with esophageal cancer in 50s
17104_000742_042522	Neuroblastoma	Unknown	1					PLCH1			Fused toes 2nd and 3rd digits bilaterally	Father adopted
17104_000177_092220	Nephroblastoma	Unknown	2					RBM26, REL			One small hyperpigmented lesion on back, ovarian cyst	Negative
17104_000162_071919	Neuroblastoma	White	2.1					ZBTB21			Negative	Negative
17104_000806_110822	Nephroblastoma	White	1.7							Trisomy 18, CN 2.99	Small and asymmetric palpebral fissures, left shorter than right, slightly prominent nasal bridge, low-set and posteriorly rotated ears, triangular helix, hypoplastic and short ears, downturned angles of mouth, side-spaced nipples, superficial cutaneous dimple with base, enlarged clitoris, camptodactyly of 2nd and 5th fingers bilaterally, broad thumbs	Maternal grandfather colon cancer
17104_000008_102219	Neuroblastoma	Asian	0.2								Negative	Negative
17104_000012_092821	Hepatoblastoma	Black or African American	0.9								Small umbilical hernia, blue spot on buttocks, failure to thrive, one small café au lait on right shoulder, neutropenia	Paternal uncles and grandfather with prostate cancer
17104_000015_071222	Nephroblastoma	White	0.5								Hemangioma at birth	Cousin with vascular anomaly, paternal grandmother with malignant melanoma
17104_000036_022020	Neuroblastoma	White	2.8								Umbilical hernia, nevus flammeus on eyelid and forehead, slow verbal development, hydrocele, freckle on back of head, infantile hemangioma on lip	Maternal great grandmother with breast cancer in 70s, paternal grandmother with stomach cancer at age 68 years, maternal great aunt with breast cancer in 40s and thyroid cancer in 60s
17104_000040_022422	Embryonal rhabdomyosarcoma	White	1.3								Nevus flammeus on forehead and back of neck, urolithiasis and left distal urethral stricture	Mother with 3-4 basal cell carcinomas starting at age 30 years. Maternal grandmother with melanoma at age 66 years, maternal great uncle with melanoma in 60s, maternal great aunt with breast cancer in 60s, maternal first cousin once removed with breast cancer at age 41 years and another one with melanoma at age 62 years, maternal great aunt with multiple myeloma, maternal great aunt with colon cancer at 60, maternal first cousin with thyroid cancer at age 68 years, maternal great grandmother with breast cancer at age 64 years
17104_000055_042619	Hepatoblastoma	Other	2.7								Beckwith-Wiedemann syndrome, large tongue at birth, hemangioma/nevus flammeus on forehead and back of neck, ear creases, previous small umbilical hernia	Sibling with stillbirth, paternal great uncle with brain tumor, and another with liver cancer at age 64 years, paternal great grandfather with liver cancer in 70s
17104_000060_081319	Malignant rhabdoid tumor	White	0.5								Small for gestational age, stork bite on back of neck	Paternal grandmother with breast cancer, paternal grandfather with kidney tumor, great aunt and great grandfather were smokers, with lung cancer
17104_000069_071019	Embryonal rhabdomyosarcoma	White	1.5								Neonatal hypoglycemia	Maternal grandmother with breast cancer at age 45 years
17104_000110_032119	Hepatoblastoma	White	2.4								Negative	Maternal great grandfather with liver cancer
17104_000116_042319	Neuroblastoma	White	0.9								Congenital absence of right thyroid lobe, autism, ventricular septal defect, macrocytosis	Maternal grandfather lymphoma at age 63 years
17104_000127_103118	Nephroblastoma	White	2.5								Negative	Maternal grandma with breast cancer at age 62 years, paternal uncles with skin cancer, and cousin with benign tumor of spinal cord at age 2 years
17104_000150_092821	Neuroblastoma	White	0.5								Stork bite on back of head	Mom with eight miscarriages before patient, maternal grandmother with ovarian tumor in her 20s, maternal greater uncle with pancreatic cancer at age 54 years
17104_000164_080619	Neuroblastoma	White	0.5								Small hemangioma on right knee	Maternal grandmother with breast cancer in 50s, maternal great aunt with breast cancer in 50s, father with leukemia at age 16 years
17104_000201_040919	Nephroblastoma	White	0.5								Bilateral Wilms, right ear crease, flat hemangioma on back of neck	Mom adopted
17104_000203_012320	Hepatoblastoma	Unknown	0.6								Extreme premie (24w), chronic lung disease, hydronephrosis, hyperinsulinemia, hypoglycemia, patent foramen ovale, inguinal hernia, right hemihypertrophy, negative Beckwith-Wiedemann testing	Negative
17104_000237_041819	Neuroblastoma	White	2.7								Negative	Cousin with retinoblastoma
17104_000254_111519	Neuroblastoma	Other	2.3								Small birthmarks on arm, leg, and neck	Maternal first cousin with neuroblastoma at age 5 years, maternal first cousin with benign tumor of hip at age 10 years, maternal great aunt with breast cancer in 50s, maternal great aunt with ovarian cancer in 50s, maternal great uncle with colon cancer in 50s, maternal great aunt with breast cancer in 40s, maternal great aunt with leukemia at age 35 years, maternal great aunt with daughter who had bone cancer at age 4 years, paternal cousin with head and neck cancer at age 18 years
17104_000264_050319	Neuroblastoma	White	1.7								Negative	Negative
17104_000285_090519	Neuroblastoma	White	0								Born with abdominal mass	Maternal grandmother with lung cancer
17104_000292_011521	Medulloblastoma	White	3.7								Palmar pits, secondary high-grade malignant glioma, frontal bossing, bilateral ovarian fibromas, basal cell carcinoma, odontogenic keratocysts, amenorrhea, hypothyroidism, short stature, myopia, unilateral sensorineural hearing loss	Paternal great aunt with ovarian cancer
17104_000332_071521	Medulloblastoma	White	6.6								Later developed sclerosing stromal tumor of ovary and pleomorphic sarcoma (location of prior radiation)	Father with thyroid cancer, mother with precancerous lesions
17104_000337_100819	Neuroblastoma	White	1								Negative	Maternal second cousin with Gorlin syndrome, maternal grandfather with basal cell carcinoma, maternal great uncle with breast cancer at age 64 years and one with lung cancer, paternal great aunt with colon cancer and great uncle with prostate cancer, grandfather with prostate cancer, paternal second cousin with medulloblastoma at age 6 years
17104_000352_101519	Neuroblastoma	Black or African American	0.8								Negative	Negative
17104_000360_032321	Hepatoblastoma	White	0.9								Neonatal hypoglycemia, large for gestational age	Negative
17104_000370_111419	Neuroblastoma	White	0.4								Red birthmark on back of neck	Maternal uncle died as infant, paternal great grandmother with lung or liver cancer at age 54 years
17104_000396_072021	Neuroblastoma	White	1.5								Mild speech delay	Maternal grandmother with breast cancer at age 49 years, maternal great grandfather with pancreatic cancer, maternal great grandmother with melanoma at age 60 years, paternal grandmother with bilateral breast cancer at age 48 years and tumors on pancreas, and ocular melanoma at age 50 years, maternal great grandmother with bilateral breast cancer, maternal great grandfather with pancreatic or prostatic cancer, maternal great great uncle with Hodgkin lymphoma at age 7 years, maternal great great aunt with melanoma in 20s and breast cancer in 30s
17104_000438_072121	Nephroblastoma	Other	4.5								Beckwith-Wiedemann syndrome, small hypopigmented lesion on abdomen, astigmatism and esotropia, right-side hydronephrosis, mild asymmetry with right arm and leg thicker than left	Maternal grandfather with prostate cancer, maternal first cousin with leukemia at age 2 years, paternal great uncle with liver cancer, and paternal cousin with thyroid cancer and one with glioma
17104_000471_021722	Neuroblastoma	White	0.7								Spitz nevus on right buttocks	Maternal grandfather with glioblastoma at age 67 years, maternal great grandmother breast cancer, maternal great aunt with ovarian cancer, and one with breast cancer twice at ages 50 and 79 years, paternal grandmother with melanoma, squamous cell carcinoma, and basal cell carcinoma in 70s. Paternal great aunts with breast cancer at age 25 years, great uncle with CLL at age 80 years, paternal great aunt with ovarian cancer
17104_000495_122120	Neuroblastoma	White	0.9								Negative	Negative
17104_000504_111920	Embryonal rhabdomyosarcoma	Unknown	2.6								Recurrent pneumonia, small birthmark on left shoulder blade (smaller than a pea, brown, not raised)	Mother, age 26 years, has a history of multiple miscarriages, colorectal polyps, and ovarian cysts, maternal great great aunt diagnosed with breast cancer at age 42 years, maternal aunt throat cancer at age 42 years (nonsmoker) and another with breast cancer diagnosed at age 52 years
17104_000536_070822	Nephroblastoma	White	2.3								Left hemihypertrophy, leg length discrepancy, precocious puberty	Paternal great aunt and uncle with multiple benign brain tumors
17104_000542_062221	Nephroblastoma	Black or African American	1.9								Top of ears folded at birth, now normal	Maternal grandmother with benign liver tumors
17104_000676_120321	Hepatoblastoma	Unknown	1.4								Negative	Negative
17104_000680_042022	Nephroblastoma	White	1.6								Negative	Father with pineal cyst *v* tumor, paternal grandfather with melanoma, paternal uncle with prostate cancer
17104_000706_022322	Hepatoblastoma	Unknown	1.5								Negative	Cousin with lymphoma
17104_000707_031622	Hepatoblastoma	White	1								Negative	Negative
17104_000750_120922	Neuroblastoma	Unknown	2.7								Negative	Negative
17104_000848_120122	Hepatoblastoma	White	2.4								Egg donor, polyhydramnios in later term, 36 hours in NICU for low O2	Maternal grandmother with breast cancer at age 69 years, and colon cancer at age 65 years
17104_000856_123022	Neuroblastoma	White	1.5								Hypoglycemia at birth	Negative
17104_000894_031523	Neuroblastoma	Unknown	0.2								IVF, one bump on ear lobe, fading birthmark on forehead and back of head	Maternal grandmother with lung cancer at age 48 years (smoker), maternal great aunt with cervical cancer, paternal great uncle with skin cancer, and great grandmother with breast cancer in 70s and sarcoma in 80s
17104_000903_022723	Nephroblastoma	Other	2.1								Bilateral Wilms	Negative
17104_000924_032823	Nephroblastoma	White	0.6								Negative	Maternal grandmother with breast cancer, paternal grandfather with bladder cancer
17104_000927_040723	Neuroblastoma	Unknown	0.9								Negative	Maternal grandmother with lymphoma
PROCA671-DNA1	Embryonal rhabdomyosarcoma	White	5.6								Hypocalcemia as an infant, large testicles	Maternal first cousin once removed with bone cancer at age 17 years, maternal great grandfather with postate cancer in 70s, father with testicular cancer at age 21 years and again at 38 years, paternal uncle with leukemia at age 17 years, paternal great uncle prostate cancer in 70s
PROCA839-DNA1	Neuroblastoma	White	0.4								Gastric foveolar hyperplastic polyps on colonoscopy and ubular adenoma, sessile serrated adenoma and inflammatory polyp. Dermal nevus with congenital features. Recent diagnosis with nodular thyroid disease	Paternal aunt with thyroid cancer in 60s
PROCA841-DNA1	Medulloblastoma	White	6.6								Congenital hip dysplasia, a few moles	Maternal great uncle with aggressive prostate cancer and melanoma, maternal grandfather with melanoma and prostate cancer in 70s, maternal aunt with cervical cancer at age 35 years, paternal great uncle with testicular cancer at age 20 years, and one with pancreatic cancer in old age
PROCA859-DNA1	Low-grade glioma	White	2.7								Hydrocele, multiple moles	Sister with cafe au lait spots, mother with benign cutaneous growths, maternal grandfather with colon cancer in 60s, father with large café au lait spot, multiple moles
PROCA866-DNA1	Medulloblastoma	White	6.3								Negative	Mother with basal cell carcinoma, maternal grandmother and grandfather with lung cancer in old age, maternal aunt with papillary thyroid cancer at age 30 years, breast cancer at age 60 years, BCC/SCC at age 50 years, paternal grandmother stomach cancer at age 73 years
PROCA873-DNA1	Teratoma	Unknown	5.6								Right back birthmark, heart murmur	Mother with history of teratomas, maternal great uncle with prostate cancer at age 69 years, maternal great aunt with breast cancer at age 70 years, maternal great grandfather at age 70 years
PROCA880-DNA1	Spindle cell sarcoma	White	0.7								Hemangioma on back of neck, small/moderate ASD	Maternal uncle with Ewing sarcoma at age 16 years, maternal grandma with inflammatory breast cancer, maternal great uncle with brain cancer, paternal great grandparents died in old age of cancers
PROCA883-DNA1	Nephroblastoma	White	3								Conceived via in vitro fertilization	Maternal great aunt with breast cancer at age 53 years, maternal great grandmother with lymphoma and lung cancer at age 80 years, maternal great grandfather with mouth cancer and skin cancer at age 80 years, paternal great grandma with colon cancer at age 75 years. Paternal great grandmother with breast cancer at age 62 years and great grandfather with skin cancer at age 60 years
PROCA890-DNA1	High-grade glioma	White	4.7								Intrauterine growth restriction	Great grandfathers with prostate and lung cancers
PROCA932-DNA1	Hepatoblastoma	White	0.9								Negative	Negative
PROCA954-DNA1	Low-grade glioma	White	6.7								Sensory processing disorder	Maternal grandmother with breast cancer and maternal great grandmother with breast cancer at age 47 years, paternal grandfather with prostate cancer at age 71 years
PROCA967-DNA1	Nephroblastoma	White	4.6								Speech delay, glasses	Maternal grandmother with endometrial cancer at age 70 years, great uncle with lung cancer and kidney cancer in 70s, maternal great uncle with gastric cancer, maternal great aunt with liver cancer, maternal great grandmother with colon cancer, paternal great uncle with prostate cancer in 70s and another with colon cancer in 70s, paternal grandfather with prostate and lung cancers in 70s
PROCA973-DNA1	Meningioma	White	8.5								Triplet, born 34w, high blood pressure	Maternal grandmother with melanoma, maternal great grandfather with lung cancer
PROCA974-DNA1	Osteosarcoma	Unknown	4.6								Negative	Maternal great grandmother with cervical and skin cancers, maternal great aunt with uterine cancer in 50s, paternal grandmother with uterine cancer in 40s and colon cancer in 60s
PROCA976-DNA1	Low-grade glioma	White	2								Negative	Maternal grandfather with colon cancer, maternal great grandmother with breast cancer, maternal great grandfather kidney cancer, paternal grandfather leukemia
PROCA981-DNA1	Osteosarcoma	White	7.4								Negative	Paternal grandfather with prostate cancer, paternal uncle with testicular cancer
PROCA984-DNA1	Hepatoblastoma	White	2.7								Negative	Father adopted
RP-2006_PROCA660-DNA1_v1_WGS_GCP	Medulloblastoma	Other	5.7								Negative	Paternal aunt with breast cancer at age 48 years, and one with benign head tumor
RP-2006_PROCA757-DNA1_v1_WGS_GCP	Hepatoblastoma	White	4.4								In vitro fertilization, trivial left ventricle dilation, aberrant origin of right vertebral artery directly from distal aortic arch and coursing posterior to esophagus	Donor egg, paternal uncle with prostate cancer in 70s
RP-2006_PROCA776-DNA1_v1_WGS_GCP	Low-grade glioma	White	5.5								Negative	Mother with papillary thyroid cancer at age 34 years, maternal uncle with testicular cancer at age 30 years, maternal grandpa with colon cancer at age 50 years, maternal great aunt with colon cancer at age 58 years, maternal great grandpa with lymphoma in 60s, father with ALL at age 13 years, paternal uncle with soft tissue sarcoma at age 14 years, paternal grandpa with polyps in mid 40s and CLL at age 63 years, paternal great grandfather with pancreatic cancer at age 51 years, paternal great grandfather with liver cancer
RP-2006_PROCA848-DNA1_v1_WGS_GCP	ATRT	White	0.4								Negative	Negative
RP-2006_PROCA907-DNA1_v1_WGS_GCP	Malignant rhabdoid tumor	White	0.9								Pyloric stenosis, red birthmark between eyes, and two dime-sized light brown birthmarks on stomach and leg	Maternal great grandmother with multiple myeloma at age 79 years, maternal great uncle with lung cancer in 30s, maternal great grandpa with lung cancer, and paternal great grandmother and great aunt with cancer

Abbreviations: ATRT, Atypical teratoid rhabdoid tumor; CPG, cancer predisposition gene; SNV, single-nucleotide variant; SV, structural variant; WGS, whole-genome sequencing.

^a^ Reflect genes found in large SV.

**TABLE A2.** Presence of Identified Constrained Gene Variants in Gabriella Miller Kids First Cancer Databases

Genes	Present Cohort			GMKF Cohorts	
	Patients, No.	Cancer Types	Concurrent Panel Identified CPG Mutation	Number of Individuals With Rare LOF Variant	Cancer Cohorts (No. of patients with mutation)
TRRAP	1	Neuroblastoma	—	32	Neuroblastoma (11), brain tumor (10), Ewing sarcoma (9), T-ALL (5), familial leukemia (4), intracranial germ cell tumor (3)
FGFR1	1	Neuroblastoma	—	30	Neuroblastoma (7), brain tumor (6), Ewing sarcoma (6), familial leukemia (5), osteosarcoma (5), T-ALL (1)
NCOA3	1	Medulloblastoma	PTCH1	24	T-ALL (7), brain tumor (4), intracranial germ cell tumor (4), neuroblastoma (4), Ewing sarcoma (3), osteosarcoma (2)
PAX6	1	Nephroblastoma	WT1	22	Brain tumor (7), neuroblastoma (6), Ewing sarcoma (4), familial leukemia (1), intracranial germ cell tumor (1), myeloid leukemia (1), osteosarcoma (1), T-ALL (1)
PTK2	1	Nephroblastoma	WT1	19	Brain tumor (6), neuroblastoma (6), T-ALL (4), Ewing sarcoma (1), myeloid leukemia (1), osteosarcoma (1)
SMARCA2	2	Nephroblastoma malignant rhabdoid tumor	—	15	Brain tumor (8), T-ALL (3), Ewing sarcoma (2), neuroblastoma (2)
ROBO2	1	Neuroblastoma	—	13	Brain tumor (2), familial leukemia (2), intracranial germ cell tumor (2), neuroblastoma (2), T-ALL (2), Ewing sarcoma (1), myeloid leukemia (1), osteosarcoma (1)
NBEA	1	Hepatoblastoma	APC	12	Brain tumor (5), T-ALL (3), Ewing sarcoma (2), neuroblastoma (2)
REST	1	Nephroblastoma	—	11	Ewing sarcoma (3), neuroblastoma (3), brain tumor (2), intracranial germ cell tumor (2), T-ALL (1)
KMT2D	1	Hepatoblastoma	—	9	Brain tumor (4), Ewing sarcoma (3), intracranial germ cell tumor (1), T-ALL (1)
CBX2	1	Neuroblastoma	—	6	Brain tumor (2), Ewing sarcoma (1), neuroblastoma (1), myeloid leukemia (1), T-ALL (1)
EIF4G1	2	Choroid plexus carcinoma mucoepidermoid carcinoma	TP53 -	5	Brain tumor (2), intracranial germ cell tumor (1), neuroblastoma (1), T-ALL (1)
MYO9A	1	Neuroblastoma	—	4	Brain tumor (1), Ewing sarcoma (1), neuroblastoma (1), T-ALL (1)
UBE2I	1	Neuroblastoma	—	2	Brain tumor (1), T-ALL (1)
DLC1	1	Hepatoblastoma	—	2	Ewing sarcoma (1), intracranial germ cell tumor (1)
HIF1A	1	Adrenal cortical carcinoma	TP53	2	Brain tumor (1), T-ALL (1)
ADAM10	1	Neuroblastoma	BRCA2	1	Brain tumor (1)

Abbreviations: CPG, cancer predisposition gene; GMKF, Gabriella Miller Kids First Data Resource Center; LOF, loss of function.

## References

[1] Wagener R, Taeubner J, Walter C, et al: Comprehensive germline-genomic and clinical profiling in 160 unselected children and adolescents with cancer. Eur J Hum Genet 29:1301-1311, 2021 33840814 10.1038/s41431-021-00878-x PMC8385053

[2] Zhang J, Walsh MF, Wu G, et al: Germline mutations in predisposition genes in pediatric cancer. N Engl J Med 373:2336-2346, 2015 26580448 10.1056/NEJMoa1508054 PMC4734119

[3] Li H, Sisoudiya SD, Martin-Giacalone BA, et al: Germline cancer predisposition variants in pediatric rhabdomyosarcoma: A report from the Children’s Oncology Group. J Natl Cancer Inst 113:875-883, 2021 33372952 10.1093/jnci/djaa204 PMC8246828

[4] Kerle IA, Gross T, Kögler A, et al: Translational and clinical comparison of whole genome and transcriptome to panel sequencing in precision oncology. NPJ Precis Oncol 9:9, 2025 39794422 10.1038/s41698-024-00788-3 PMC11724059

[5] Amoretti M, Amsler C, Bonomi G, et al: Production and detection of cold antihydrogen atoms. Nature 419:456-459, 2002 12368849 10.1038/nature01096

[6] Huang K-L, Mashl RJ, Wu Y, et al: Pathogenic germline variants in 10,389 adult cancers. Cell 173:355-370.e14, 2018 29625052 10.1016/j.cell.2018.03.039 PMC5949147

[7] Mirabello L, Zhu B, Koster R, et al: Frequency of pathogenic germline variants in cancer-susceptibility genes in patients with osteosarcoma. JAMA Oncol 6:724-734, 2020 32191290 10.1001/jamaoncol.2020.0197 PMC7082769

[8] Tate JG, Bamford S, Jubb HC, et al: COSMIC: The catalogue of somatic mutations in cancer. Nucleic Acids Res 47:D941-D947, 2019 30371878 10.1093/nar/gky1015 PMC6323903

[9] Karczewski KJ, Francioli LC, Tiao G, et al: The mutational constraint spectrum quantified from variation in 141,456 humans. Nature 581:434-443, 2020 32461654 10.1038/s41586-020-2308-7 PMC7334197

[10] Desrosiers-Battu LR, Wang T, Reuther J, et al: Comparing the diagnostic yield of germline exome versus panel sequencing in the diverse population of the Texas KidsCanSeq Pediatric Cancer study. JCO Precis Oncol 10.1200/PO.24.00187 10.1200/PO.24.00187 PMC11392521 39259914

[11] Gillani R, Collins RL, Crowdis J, et al: Rare germline structural variants increase risk for pediatric solid tumors. Science 387:eadq0071, 2025 39745975 10.1126/science.adq0071 PMC12873715

[12] Schneppenheim R, Frühwald MC, Gesk S, et al: Germline nonsense mutation and somatic inactivation of SMARCA4/BRG1 in a family with rhabdoid tumor predisposition syndrome. Am J Hum Genet 86:279-284, 2010 20137775 10.1016/j.ajhg.2010.01.013 PMC2820190

[13] Wegert J, Ishaque N, Vardapour R, et al: Mutations in the SIX1/2 pathway and the DROSHA/DGCR8 miRNA microprocessor complex underlie high-risk blastemal type Wilms tumors. Cancer Cell 27:298-311, 2015 25670083 10.1016/j.ccell.2015.01.002

[14] Mahamdallie SS, Hanks S, Karlin KL, et al: Mutations in the transcriptional repressor REST predispose to Wilms tumor. Nat Genet 47:1471-1474, 2015 26551668 10.1038/ng.3440

[15] Stoltze UK, Foss-Skiftesvik J, Hansen TO, et al: The evolutionary impact of childhood cancer on the human gene pool. Nat Commun 15:1881, 2024 38424437 10.1038/s41467-024-45975-9 PMC10904397

[16] Roy A, Sakthikumar S, Kozyrev SV, et al: Using evolutionary constraint to define novel candidate driver genes in medulloblastoma. Proc Natl Acad Sci USA 120:e2300984120, 2023 37549291 10.1073/pnas.2300984120 PMC10438395

